# Breast-conserving surgery with 3D-printed surgical guide: a single-center, prospective clinical study

**DOI:** 10.1038/s41598-021-81936-8

**Published:** 2021-01-26

**Authors:** Zhen-Yu Wu, Hee Jeong Kim, Jongwon Lee, Il Yong Chung, Jisun Kim, Saebyeol Lee, Byung Ho Son, Sei-Hyun Ahn, Hak Hee Kim, Joon Beom Seo, Jae Ho Jeong, Gyungyub Gong, Namkug Kim, BeomSeok Ko

**Affiliations:** 1 Division of Breast Surgery, Department of Surgery, University of Ulsan College of Medicine, Asan Medical Center, 88, Olympic-ro 43-gil, Songpa-gu, Seoul, 05505 Republic of Korea; 2 Biomedical Engineering Research Center, Asan Institute for Life Sciences, Asan Medical Center, Seoul, Korea; 3 Department of Breast Surgery, Shanghai East Hospital, Tongji University School of Medicine, Shanghai, China; 4 Department of Radiology, Convergence Medicine, University of Ulsan College of Medicine, Asan Medical Center, 88, Olympic-ro 43-gil, Songpa-gu, Seoul, 05505 Republic of Korea; 5 Department of Oncology, University of Ulsan College of Medicine, Asan Medical Center, Seoul, Korea; 6 Department of Pathology, University of Ulsan College of Medicine, Asan Medical Center, Seoul, Korea; 7 Department of Convergence Medicine, University of Ulsan College of Medicine, Asan Medical Center, Seoul, Korea

**Keywords:** Breast cancer, Surgical oncology

## Abstract

To facilitate precise tumor resection at the time of breast-conserving surgery (BCS), we developed and implemented a magnetic resonance imaging (MRI)-based three-dimensional-printed (3DP) breast surgical guide (BSG). This prospective cohort study was conducted at a single institution from July 2017 to February 2019 on women with breast cancer who underwent partial breast resection using patient-specific 3DP BSGs. Eighty-eight patients with invasive cancer were enrolled, of whom 1 patient had bilateral breast cancer. The mean size of the tumor long-axis on MRI before surgery was 2.8 ± 0.9 cm, and multiple tumors were observed in 34 patients. In 16 cases (18.0%), the resection margin was tumor-positive according to intraoperative frozen biopsy; all of these tumors were ductal carcinoma in situ and were re-excised intraoperatively. In 93.3% of the cases, the resection margin was tumor-free in the permanent pathology. The mean pathological tumor size was 1.7 ± 1.0 cm, and the mean distance from the tumor to the border was 1.5 ± 1.0 cm. This exploratory study showed that the tumor area on the MRI could be directly displayed on the breast when using a 3DP BSG for BCS, thereby allowing precise surgery and safe tumor removal.

*Trial Registration* Clinical Research Information Service (CRIS) Identifier (No. KCT0002375, KCT0003043).

## Introduction

For early breast cancer, breast-conserving surgery (BCS) is recommended, as BCS and mastectomy are associated with similar survival outcomes^1^. It is important to accurately predict the extent of the tumor before surgery because tumor involvement in the resection margin during partial breast resection is closely related to recurrence and prognosis^2^.

Imaging techniques, such as mammography, ultrasonography, and magnetic resonance imaging (MRI), are commonly used to determine the preoperative tumor extent to facilitate precise BCS. Several localization techniques have been used to indicate the extent of tumors that are difficult to palpate^3^. MRI is known to be more sensitive than mammography or ultrasonography in quantitatively identifying tumor regions^4^. However, conventional tumor localization methods have limitations in using MRI data to directly mark the range of tumor resection in current clinical practice^5,6^. The development of new tumor localization techniques that can overcome these limitations is imperative to improve the precision of BCS as well as local control. Therefore, we developed a breast surgical guide (BSG) using three-dimensional printing and prospectively investigated patients who underwent BCS using a three-dimensional-printed (3DP) BSG based on supine MRI.

## Methods

### Eligibility

In this single-center, prospective cohort study, women fulfilling the following criteria were eligible for enrollment: age 18–69 years with confirmed breast cancer and available for radiological and physical evaluations after BCS. Patients with multiple lesions were eligible. Patients with contraindications for MRI or those who underwent neoadjuvant systemic therapy were excluded. This study was approved by the Institutional Review Board of Asan Medical Center (Nos. 2016-1237, 2018-0690) and performed in accordance with the principles of the Declaration of Helsinki. Patients provided informed consent and agreed to the addition of a supine series to the standard baseline MRI protocol.

### Production of the surgical guide

**Figure 1:**
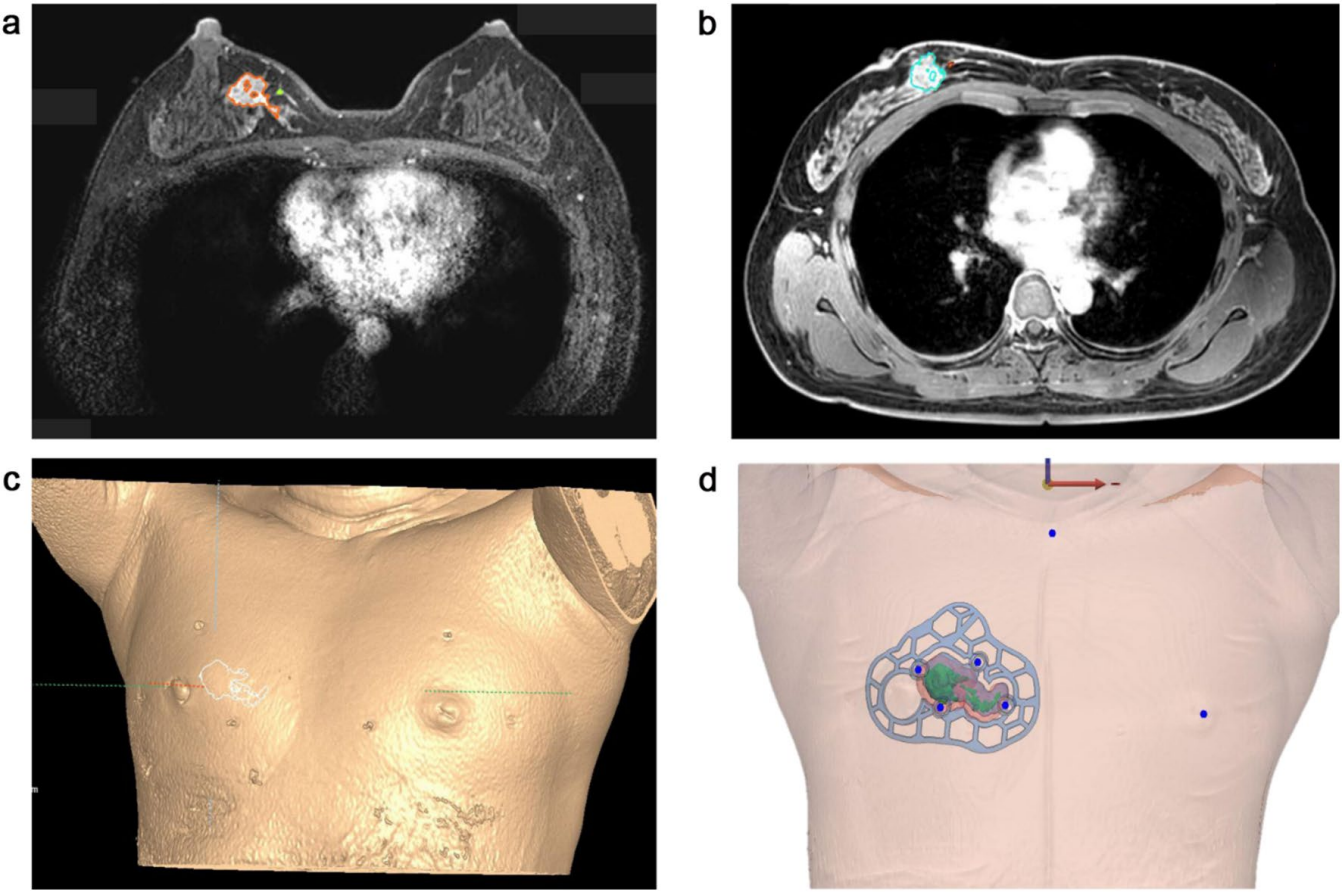
Segmentation of the breast and tumor in prone/supine magnetic resonance imaging (MRI) for breast surgical guide (BSG) modeling. (**a**) Segmented tumor on prone MRI, (**b**) Segmented tumor on supine MRI, (**c**) 3D modeling of the breast and tumor on supine MRI, (**d**) BSG tailored to the patient's breast surface to target tumors inside the breast.

Breast images were acquired using a 3.0 T MRI system (Ingrain; Philips Healthcare, Netherlands) and breast coil. To obtain the same posture as used in operation, both arms were raised and additional magnetic resonance images were obtained with the patient in the supine position. The standard breast MRI protocol included a T2 spectral-attenuated inversion recovery, a T1 without fat suppression, a short-tau inversion recovery sequence, a diffusion-weighted MRI, and a dynamic perfusion study with intravenous injection of 0.1 mmol/kg of gadopentetate dimeglumine (MultiHance, Gd-BOPTA; Bracco Imaging SpA, Milan, Italy), followed by flushing with 20 ml of saline solution at 2 ml/s^7^. The dynamic study involved one pre-contrast acquisition followed by five post-contrast acquisitions of T1-weighted high-resolution volume examinations^7^. Immediately after the acquisition of the last dynamic series, the patient was removed from the magnet, the breast coil was removed, and the patient was invited to assume the supine position^7^. A multi-point Dixon sequence was used for MRI acquisition in the supine position^7^. Breast tissue and tumor were segmented from imaging data obtained from prone/supine MRI using Mimics Medical v17 (Materialise Inc., Belgium) (Fig. 1a–c). The BSG was modeled with a 0.5 cm distance from the tumor boundary to guarantee a safety margin. The following specifications were used in the modeling of the BSG to ensure accurate display of the tumor resection boundary: (1) fit to the breast skin surface, (2) a hole to fit the nipple, and (3) guidelines to prevent the rotation of the BSG and indicate the opposite nipple and suprasternal notch. The BSG was manufactured as a hybrid type with a groove that can be marked on the skin surface and a column that can be targeted by injecting blue dye around the tumor located inside the breast (Fig. 1d). The column also has an anchor, to which a 1 cc syringe can be attached. The anchor depth can then be customized according to the MRI evaluation so that the injection depth can be set.

### Operation and pathologic assessments

**Figure 2:**
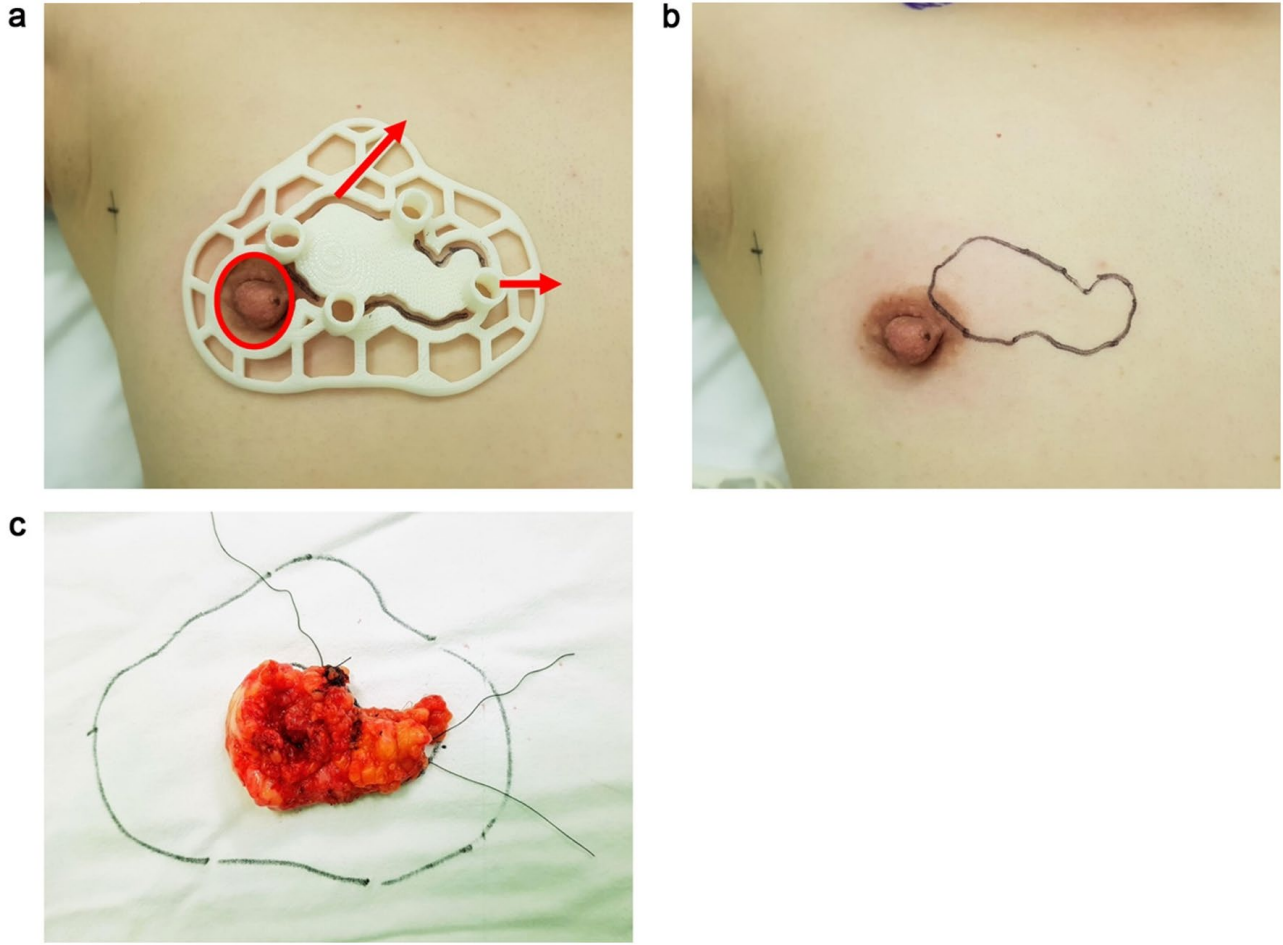
Partial breast excision by applying a breast surgical guide (BSG). (**a**) To accurately localize the tumor, fix the BSG based on both nipples and the suprasternal notch, (**b**) Quantitatively mark the area of the tumor on the breast surface along the groove of the BSG, (**c**) Match the removed breast specimen to the tumor area in the BSG.

Patient-specific BSGs were printed and sterilized preoperatively. For accurate tumor targeting, the nipple was inserted into the hole of the BSG, and the BSG was fixed using a reference line pointing to the suprasternal notch and the nipple on the opposite side (Fig. 2a). A tumor resection boundary was drawn along the line designed to match the tumor shape (Fig. 2b). Blue dye was injected through the column to indicate the extent of removal around the tumor. After removing the tumor based on the blue dye in the breast tissue, the tumor was matched with the shape of the BSG to determine whether further removal was required (Fig. 2c). To check for residual cancer, tissue was removed from several cavity sites, and frozen biopsy was performed. Re-excision was performed when residual tumor was detected. The absence of continuity in the breast tissue was considered to have no effect on recurrence and was not considered in the evaluation of the resection margin^8^. The distance from the tumor edge to the resection margin was measured in the 3, 6, 9, and 12 o'clock directions. Sentinel lymph node biopsy (SLNB) was performed according to cancer type, and axillary lymph node dissection (ALND) was performed according to the presence of node metastasis.

### Endpoints and statistical analysis

The primary endpoints were the proportion of tumor-free resection margins, intraoperative frozen section results, permanent pathology results after surgery, and the distance from the tumor to the margins for ensuring proper removal. In univariate analysis of factors associated with positive resection margins, the Chi-square or Fisher’s exact test was used to compare differences between subgroups. All statistical analyses were performed using IBM SPSS Statistics software version 24.0 for Windows (IBM Corp., Armonk, NY). A 2-sided *P* < 0.05 indicated statistical significance.

## Results

Table 1 describes the patient and tumor characteristics. From July 2017 to February 2019, 88 patients with invasive cancer were enrolled in the study, of whom 1 patient had bilateral breast cancer. The median age of the patients was 48 years (range 23–69 years). The mean size of the tumor long axis on MRI performed before surgery was 2.8 ± 0.9 cm, and multiple tumors were observed in 34 patients. Most tumors (n = 83, 93.3%) were removed with an adequate surgical margin upon final pathological evaluation (Table 2). In 16 cases (18.0%), the resection margin was tumor-positive according to intraoperative frozen biopsy; all of these tumors were ductal carcinoma in situ (DCIS) and were re-excised intraoperatively. There were no cases with conversion to mastectomy, and the final pathologic results showed tumor negativity in the margin. In 7 cases, metastatic axillary lymph nodes were found, and ALND was performed. In 1 case of small DCIS diagnosed with preoperative needle biopsy, axillary surgery was not performed. The mean diameter of the tumor long axis was 1.7 ± 1.0 cm and that of the long axis of the removed specimen was 5.7 ± 1.9 cm. The mean distance between the tumor and resection margin was 1.5 ± 1.0 cm. Among the patients who underwent SLNBs only as axillary surgery, the median operation time was 75 min (range 44–167 min), compared with 89 min (range 71–114 min) among patients underwent ALND. Table 3 shows the univariate analysis of factors associated with positive margins in frozen/permanent biopsies. Among variables such as tumor histotype, multifocality, presence of an intraductal component, tumor diameter, molecular subtype, and nearest margin status, the presence of an intraductal component was the only factor statistically significantly associated with margin positivity.

**Table 1:** Patient and tumor characteristics.

Variables	N (%)
**Age (years)**	
Median	48
Range	23–69
< 50	50 (56.2)
≥ 50	39 (43.8)
**Pathology**	
Invasive ductal carcinoma	73 (82.0)
Invasive lobular carcinoma	5 (5.6)
Mixed	5 (5.6)
Others	6 (6.7)
**T stage**	
T1	63 (70.8)
T2	25 (28.1)
T3	1 (1.1)
**N stage**	
N0	68 (76.4)
N1	18 (20.2)
N2	3 (3.4)
**Histologic grade**	
I	4 (4.5)
II	68 (76.4)
III	17 (19.1)
**Multifocality**	
Yes	34 (38.2)
No	55 (618)
**Subtype**	
HR +/HER2 −	65 (73.0)
HR +/HER2 +	14 (15.7)
HR −/HER2 +	3 (3.4)
TN	7 (7.9)
**Lymph node status**	
Negative	68 (76.4)
Positive	21 (23.6)
**Axillary surgery**	
SLNB only	82 (92.1)
ALND	7 (7.9)

*SLNB* sentinel lymph node biopsy, *ALND* axillary lymph node dissection, *HR* hormone receptor, *HER2* human epidermal growth factor receptor 2, *TN* triple negative.

**Table 2:** Characteristics of the surgical specimens.

Variables	N (%)
**Margin status, frozen section**	
Negative	73 (82.0)
Positive	16 (18.0)
**Margin status, permanent section**	
Negative	83 (93.3)
Positive	6 (6.7)
**Operation time (min)**	
Mean ± SD	78 ± 19
SLNB only-median	75
SLNB only-range	44–167
ALND-median	89
ALND-range	71–114
**Nearest margin (cm)**	
Mean ± SD	0.8 ± 0.5
Median	0.7
Range	0.1–2.0
**Tumor diameter (cm)**	
Mean ± SD	1.7 ± 1.0
Median	1.5
Range	0.2–6.5
**Specimen diameter (cm)**	
Mean ± SD	5.7 ± 1.9
Median	5.3
Range	3.0–12.0
**Tumor-to-margin distance (cm)**	
Mean ± SD	1.5 ± 1.0
Median	1.5
Range	0.1–7.0

*SD* standard deviation.

**Table 3:** Univariate analysis of risk factors for positive margins in frozen/permanent biopsies.

Variables	Positive margin		Clear margin		*P* value
	N	%	N	%	
**Histotype**					
Invasive ductal carcinoma	12	16.4	61	83.6	0.07
Invasive lobular carcinoma	3	60.0	2	40.0	
Mixed	2	40.0	3	60.0	
Others	2	33.3	4	66.7	
**Multifocality**					
Yes	10	29.4	24	70.6	0.144
No	9	16.4	46	83.6	
**Intraductal component**					
Present	11	35.5	20	64.5	0.017
Absent	8	13.8	50	86.2	
**Tumor diameter (cm)**					
≤ 2	15	23.8	48	76.2	0.57
> 2	4	15.4	22	84.6	
**Molecular subtype**					
HR + /HER2 −	16	24.6	49	75.4	0.604
HR + /HER2 +	2	14.3	12	85.7	
HR-/HER2 +	0	0.0	3	100.0	
TN	1	14.3	6	85.7	
**Nearest margin (cm)**					
< 1	12	21.4	44	78.6	0.764
≥ 1	6	18.8	26	81.3	

*HR* hormone receptor, *HER2* human epidermal growth factor receptor 2, *TN* triple negative.

## Discussion

In early-stage breast cancer, BCS followed by whole breast radiotherapy is the standard protocol for local treatment and is as safe as mastectomy^1^. For optimal cosmetic results, normal tissue should be preserved as much as possible, but a clear resection margin should be obtained because positive margins increase the risk of recurrence^9^. With nonpalpable tumors or tumors with unclear borders, resection margins are often tumor-positive, and the rate of margin positivity is reported to be 14–47%^9^. Mammography, ultrasonography, and MRI are used to identify the tumor extent, and localization procedures are required to accurately target nonpalpable tumors and obtain clear margins. Wire-guided localization (WL) has been used as a standard technique for patients with nonpalpable tumors undergoing BCS^11^. Although WL is simple, it causes pain and various complications, such as cutting, migration, and pneumothorax, and the wire may be lost during surgery^12^. The greatest limitation of WL is that it cannot accurately represent the tumor extent. To overcome the limitations of WL, new targeting techniques have been tried, including radioactive seed localization, nonradioactive localization, and magnetic seeds. However, these techniques are limited by radiation exposure, poor initial seed placement, seed displacement, depth limitation, and high cost. Additionally, there is a limitation related to the difficulty of marking the extent of breast tumors, as depicted by imaging^10,13–24^ (Table 4).

**Table 4:** Tumor localizations and lumpectomy techniques in breast-conserving surgery.

Localization technique	Clear margin rate	Original extension	Using MRI (quantitative )	No pain	No procedure time	No radiation	No material movement
BSG	82–93.3% this article	O	O	O	O	O	O
WL	72.7–88.6%^10,15–17^	X	△	X	X	△	△
Carbon	85%^18^	X	X	X	X	△	△
Clip	85.9–92%^19,20^	△	X	X	X	△	△
RSL	69.7–97%^10,15,16,21^	△	X	X	X	X	△
IOUS	76–100%^17,22^	O	X	O	X	O	O
Cavity shave	81–90.3%^23,24^	X	X	O	X	O	O

*BSG* breast surgical guide, *WL* wire localization, *carbon* carbon marking, *clip* clip marker localization, *RSL* radioactive seed localization, *IOUS* intraoperative ultrasound-guided excision, *time* procedure time.

MRI defines the tumor extent more accurately than other imaging modalities, and it is known to accurately detect multifocal or multicentric cancers^25^. However, it is difficult to precisely mark the MRI-determined tumor extent directly on the breast. Sakakibara et al.^6^ used supine MRI for patients with DCIS and a projection technique to indicate the extent of the breast tumor for performing BCS. Matthew et al.^26^ compared the size and location of tumors using prone and supine MRI and optical scans. However, these methods are not routinely performed due to the potential inaccuracy of targeting or difficulty of the procedure. Barth et al.^27^ reported that patient-specific 3DP forms based on supine MRI could be used safely and accurately to remove tumors using BCS. In patients who receive neoadjuvant systemic therapy, it is difficult to remove the tumor precisely because the pretreatment primary tumor margins cannot be confirmed using conventional methods, and excessive removal of normal tissues occurs frequently.

In a previous pilot study, we confirmed that tumors in patients receiving neoadjuvant chemotherapy can be safely removed using BSG^28^. Theoretically, when using BSGs, the tumor should be precisely and completely removed. In this current prospective study, 6.7% of cases involved a positive margin in the final pathology. It is accepted that the real tumor extent is larger than the MRI-determined tumor extent, and positive margins are observed due to an inherent limitation of MRI. In another prospective clinical trial conducted at our institution^29^, we employed an MRI-based 3DP BSG for BCS in patients with DCIS^29^. Although a relatively small number of patients were involved in that BSG series, pathologically clear resection margins were obtained in all patients^29^. DCIS lesions are generally nonpalpable and are frequently associated with a diffuse growth pattern. Further, they sometimes cover areas larger than the visible extent of the lesion on preoperative imaging^29^. These features make it difficult to accurately determine and localize the exact extent of the tumor before surgery, thus potentially resulting in frequent margin positivity and re-excision. However, the preliminary results of our previous study showed the effectiveness of this device in patients with DCIS^29^. Notably, in this study, the presence of an intraductal component was observed as the only significant factor associated with positive resection margins. This result highlights the importance of a precise tumor localization strategy in patients with DCIS component.

This study was limited by its single-institution nature, the small single-arm sample size, the volume of the removed tissue as well as the fact that the cosmetic results were not evaluated. A future large-scale controlled study is still needed to further validate the accuracy and effectiveness of the 3DP BSG in breast cancer patients.

In conclusion, the application of a supine MRI-based 3DP BSG in BCS safely removed tumors with low rates of positive margins. BSG has several advantages over the existing methods used for localization, and in particular, it has the advantage of providing quantitative information about the area of the tumor.
